# Effective Therapeutic Delivery and Bioavailability Enhancement of Pioglitazone Using Drug in Adhesive Transdermal Patch

**DOI:** 10.3390/pharmaceutics11070359

**Published:** 2019-07-23

**Authors:** Anroop B. Nair, Sumeet Gupta, Bandar E. Al-Dhubiab, Shery Jacob, Pottathil Shinu, Jigar Shah, Mohamed Aly Morsy, Nagaraja SreeHarsha, Mahesh Attimarad, Katharigatta N. Venugopala, Sabah H. Akrawi

**Affiliations:** 1 Department of Pharmaceutical Sciences, College of Clinical Pharmacy, King Faisal University, Al-Ahsa 31982, Saudi Arabia; 2 Department of Pharmacology, M. M. College of Pharmacy, Maharishi Markandeshwar (Deemed to be University), Mullana 133203, India; 3 Department of Pharmaceutical Sciences, College of Pharmacy, Gulf Medical University, Ajman 4184, UAE; 4 Department of Biomedical Sciences, College of Clinical Pharmacy, King Faisal University, Al-Ahsa 31982, Saudi Arabia; 5 Department of Pharmaceutics, Institute of Pharmacy, Nirma University, Ahmedabad 382481, Gujarat, India; 6 Department of Pharmacology, Faculty of Medicine, Minia University, El-Minia 61511, Egypt; 7 Department of Biotechnology and Food Technology, Durban University of Technology, Durban 4000, South Africa

**Keywords:** Duro-Tak, flux, permeation enhancer, pharmacokinetics, rat, release

## Abstract

The administration of pioglitazone as an oral therapy is restricted due to various challenges. The aim of the current investigation was to evaluate the suitability of pioglitazone in adhesive transdermal patch as an alternative delivery system, in order to improve therapeutic delivery. Drug in adhesive pioglitazone (2% *w*/*w*) transdermal patch were optimized for drug release, suitable adhesive, and skin permeation enhancer. The selected patch was examined for drug-loading capacity and the patch with greater pioglitazone (6% *w*/*w*) was evaluated in rat models. The release of pioglitazone was influenced by the tested adhesive and was shown to be significantly higher (*p* < 0.001) with patch, prepared using Duro-Tak 87-2516. The ex vivo permeation results substantiate the release data as a greater transdermal flux (15.67 ± 2.35 µg/cm^2^/h) was demonstrated in patch fabricated with Duro-Tak 87-2516. Skin penetration enhancers promoted the ex vivo transdermal delivery of pioglitazone, and was ~2 folds (*p* < 0.0001) higher with propylene glycol, as compared to patch without enhancer. The maximum solubility of pioglitazone in Duro-Tak 87-2516 was found to be 6% *w*/*w*. Increasing the drug content in patch enhanced the transdermal flux and was highest when the pioglitazone level was 6% *w*/*w* (72.68 ± 5.76 µg/cm^2^/h). In vivo pharmacokinetic data demonstrate that the AUC_0-α_ in transdermal application (13,506.51 ± 1649.92 ng·h/mL) was ~2 times higher (*p* < 0.0001) as compared to oral dosage form. In conclusion, the promising results observed here signifies that developed patch could be a viable alternative for oral therapy of pioglitazone.

## 1. Introduction

Diabetes is currently recognized as one of the global epidemics by the World Health Organization. The prevalence of diabetes has increased dramatically in the last decade and is growing quickly in low income countries [1]. It is a chronic, progressive disorder and is associated with clinical complications, including microvascular diseases, such as cardiopathy, nephropathy, retinopathy, and macrovascular diseases, which cause blindness, cardiovascular diseases, kidney impairment, and death if left uncontrolled. Among diabetes, Type 2 diabetes is accountable for ~95% of conditions that are significantly linked with older age, genetic disposition, obesity, and physically inactive lifestyle [2]. Many categories of hypoglycemic agents are presently used in the management of type 2 diabetes, which acts through a diverse mechanism of actions, such as in promoting insulin secretion, minimizing insulin resistance, or improving insulin penetration into the cells [3]. Regardless of the availability of different types of medications, the management of type 2 diabetes still remains complex [4]. Most of these drugs also possess several potential side effects and are not limited to hepatotoxicity, low blood sugar, weight gain, dizziness, kidney complications, and diarrhea [5].

Glitazones are the new categories of insulin sensitizers, which are currently preferred over other hypoglycemic agents for Type 2 diabetes therapy, because of their positive effects on glucose level, insulin sensitivity, and the cardiometabolic profile [6]. Indeed, glitazones improve insulin resistance and reduce macrovascular risk in patients with diabetes, and specifically target muscular insulin resistance [7]. Pioglitazone is a thiazolidinedione derivative currently indicated in the management of type 2 diabetes [8]. The potential of pioglitazone in other treatments like cancer [9], Alzheimer’s [10], inflammation [11], wound healing [12] were also established. However, the updated drug safety communication review report from the US Food and Drug Administration (FDA) suggests that the labels of all pioglitazone, containing medicines, should have a warning about the increased risk of bladder cancer [13]. Pioglitazone is effective in significantly lowering glycosylated hemoglobin (HbA(1c)), decreasing fasting and post-prandial plasma glucose levels, and improves beta-cell function [14]. Pioglitazone demonstrates a lower advancement rate of cardiac complications, due to the superior cardioprotective benefits and is more suitable for diabetic dyslipidemic population [15]. The pharmacokinetics of pioglitazone after oral administration of a single dose (30 mg) in humans showed maximum serum concentrations (C_max_) of 900 ng/mL and total drug concentration of about 8000 ng/mL along with short half-life (3–6 h) [16]. The oral therapy of pioglitazone is limited due to various associated side effects, including potentially serious conditions, such as hepatotoxicity and, therefore, routine liver function tests are suggested in patients [17]. Furthermore, low aqueous solubility of pioglitazone (1.8 µg/mL) has limited its therapeutic efficacy in oral therapy [18]. Various approaches have been assessed to enhance the therapeutic delivery of pioglitazone, including vesicular carrier systems, such as microscapsules [19], nanoparticles [20], as well as gastro retentive drug delivery systems [21], and self-emulsifying drug delivery systems [22].

Transdermal drug administration is a viable alternative to conventional oral therapy and provides incessant delivery of actives to the systemic circulation like intravenous injections [23]. The transdermal therapy has the potential to bypass the first pass metabolism, as well as decreases gastrointestinal side effects and, thereby, improve the safety and patient adherence. However, the transdermal delivery systems are not suitable for drugs with large molecular weight or which causes skin sensitization or irritation [24]. In general, the physicochemical properties of drug molecules, including aqueous solubility (>1 mg/mL), molecular size (<500 Daltons), lipophilicity (log P of 1–3), melting point (<200 °C), and dose (<25 mg/day) are considered, while developing transdermal drug delivery systems [25]. Accordingly, conventional transdermal systems are suitable for small, lipophilic and low dose drugs, while they fail to deliver macromolecules. Therefore, both physical and chemical approaches have been proposed and developed to improve the transdermal flux using electricity [26], ultrasound [27], heat [25], microneedle [28], chemical enhancers [25], bio-functional textiles [29], as well as carrier systems like micro/nano-emulsions [30], elastic ultra-deformable vesicles including liposomes, transfersomes, invasomes, ethosomes, and niosomes [31]. In the above context, the physicochemical properties of pioglitazone like small molecular weight (356 Da), good partition coefficient (log P 2.94), low melting point (194 °C), and suitable pKa (5.8) are ideal for the transdermal delivery. Moreover, transdermal system of pioglitazone can be potentially beneficial in reducing adverse effects and maintaining optimum plasma drug level, which in turn could effectively manage blood sugar. Investigations have been carried out to deliver pioglitazone through the transdermal route using nanostructured lipid carriers [32], as well as transgel system, constituted of proniosomes/niosomes [33]. However, being a biopharmaceutical class II compound, the major issue concerning pioglitazone is its poor aqueous solubility. In this background, a suitable drug delivery system which can load higher amount of pioglitazone and release it on the skin surface is ideal for its transdermal therapy. Formulations like hydrogels (porous/nonporous) may not be suitable because a higher drug loading of hydrophobic pioglitazone is usually not possible. Alternatively, drug in adhesive (DIA) transdermal system could be more appropriate as it would assist high drug loading and thereby more drug delivery.

Typically, the DIA transdermal system is a miniaturized patch that is applied over the skin, which eventually leads to prolonged percutaneous delivery of active substances. This passive delivery approach provide several advantages, including adequate skin adhesion, easy to formulate, good physical integrity, high permeability, controlled drug delivery, no dose dumping, and higher patient compliance [34]. DIA patch fabricated using acrylic pressure sensitive adhesives has established it’s prospective as an efficient transdermal drug delivery system [35]. The essential components in these systems are acrylic pressure sensitive adhesive, backing membrane and a release liner. Thus, the DIA systems are typically evaluated for suitable adhesive, drug content, as well as the transdermal permeation enhancers. Indeed, acrylic pressure sensitive adhesives allow the incorporation of drug and permeation enhancers into it, provides close contact with the skin and, thereby, enhance the drug partitioning into the skin. Accordingly, the aim of the current investigation was to develop DIA pioglitazone patch for the transdermal therapy and evaluate its delivery both in vitro and in vivo. DIA pioglitazone system was fabricated and investigated the effect of various formulation constituents like acrylic pressure sensitive adhesives, skin permeation agents, and the solubility of pioglitazone. The selected DIA pioglitazone patch was further evaluated in vivo by assessing the pharmacokinetics in rats.

## 2. Materials and Methods

### 2.1. Materials

Pioglitazone was donated by Ind-Swift Ltd., Parwanoo, Himachal Pradesh, India. Diethylene glycol monoethyl ether, *N*-methyl-2-pyrrolidone (NMP), Tween 80, oleic acid and propylene glycol were purcahsed from Sigma Aldrich, St. Louis, MO, USA. Duro-Tak 87-4098, Duro-Tak 87-900A, Duro-Tak 87-9301, Duro-Tak 87-4287, Duro-Tak 87-2516 were obtained from the National Starch and Chemical Company, Bridgewater, NJ, USA. Release liner (Scotchpak^®^ 1022) and backing membrane (CoTran^TM^ 9720) were obtained from 3M, St. Paul, MN, USA.

### 2.2. Estimation of Pioglitazone

The amount of pioglitazone in samples was determined using high-performance liquid chromatography (HPLC) system (LC-10ATVP; Shimadzu Corporation, Tokyo, Japan). Chromatographic separation of pioglitazone was performed on zorbax C18 column (150 mm × 4.6 mm i.d, 5 µm). The mobile phase consists of acetonitrile, 0.1 M ammonium acetate, and glacial acetic acid (25:25:1) with an injection volume of 50 µL. The flow rate of 1.0 mL/min was used to elute pioglitazone isocratically at 25 °C, and was detected at 265 nm. The validated method has a retention time of 5.8 min with a linearity in the range of 20–1000 ng/mL (*r*^2^ > 0.991).

### 2.3. Formulation of DIA Patches

The first step in developing an adhesive transdermal patch is the selection of a suitable acrylic adhesive. Following adhesives (Duro-Tak 87-2516, Duro-Tak 87-4287, Duro-Tak 87-4098, Duro-Tak 87-900A and Duro-Tak 87-9301) were chosen to formulate pioglitazone transdermal patches. DIA pioglitazone patches were fabricated by solvent evaporation method. Briefly, required amount of pioglitazone was uniformly mixed with adhesives. The homogenous mixture was placed on the Scotchpak^®^ 1022 and the thickness was ~1500 µm. The patches were dried in a hot air oven at 40 °C (2 h). Afterwards the patches were cut to circular disc and CoTran^TM^ 9720 was fixed on the cast layer by a hand roller. Similarly, patches were prepared with skin permeation enhancers (diethylene glycol monoethyl ether, NMP, oleic acid, PG and Tween 80) by incorporating adequate quantity.

### 2.4. Drug Content in Patch

The pioglitazone content in developed DIA patches was quantified in an area of 1 cm^2^. The patch was placed in mobile phase and sonicated in a sonicator bath (Model No. 8890, Cole-Parmer, Niles, IL, USA) for a period of 1 h and centrifuged (12,000 rpm for 5 min). The pioglitazone content was determined after filtering the supernatant solution using HPLC.

### 2.5. In Vitro Release

The release of pioglitazone from prepared DIA patches was evaluated by paddle over disc method using USPXXIV Type II apparatus (Electro Lab TDC 50, Mumbai, India). Patch with size measuring 1.5 cm × 1.5 cm was cut and attached on a teflon disc. The whole assembly was kept in the dissolution vessel so that the drug loaded surface of patch was exposed towards the dissolution medium. Phosphate buffer (500 mL; pH 6) with 10% Tween 80 was used as the dissolution medium, temperature was maintained at 32 ± 0.5 °C and the paddle was rotated at 50 rpm. Samples were taken at specific time periods, filtered using syringe membrane filter (0.2 μm, Millipore Corporation, Bedford, MA, USA) and readily analyzed by HPLC. A control experiment was carried out with pure drug. The in vitro release data was assessed using various mathematical models. The model, which showed a high correlation coefficient (*r*^2^) value for the release data, is considered as the best fit.

### 2.6. Ex Vivo Permeation

Among rodent models, the rat skin is mostly used in permeation studies due to its availability, ease of handling, small size, and less expensive [36], though more permeable than the human skin, influence of physicochemical properties on the skin transport can be effectively determined using the rat skin [37]. Wistar rats were sacrificed to get the full thickness skin membrane. The hairs were removed by trimming with an electric clipper. The subdermal tissue was removed using scissors and stored at −20 °C in a deep freezer until further investigation. The skin was thawed and the resistance was determined before the permeation experiments [38]. The skin membrane which had resistance of >20 kΩ.cm^2^ was used for experimental investigations. The transport of pioglitazone across the rat skin was measured for 12 h utilizing vertical Franz diffusion cell (Logan Instruments Ltd., Somerset, NJ, UK). The excised skin was held between two compartments with the dermis region in contact with receptor fluid containing phosphate buffered saline (pH 7.4) with 10% Tween 80 (PBS-T). The total available surface area for drug transport was 0.64 cm^2^. PBS-T fluid was placed in both donor (1 mL) and receptor (5 mL) compartments for equilibration. After this step, donor and receptor fluid were discarded from the individual chambers and a circular DIA patch (0.6 cm^2^) was fixed by exercising a mild pressure to fix the system to the skin. Fresh PBS-T was added in the receptor compartment and mixed at constant rpm (600 rpm) and the temperature was held at 37 ± 0.5 °C by a circulating water bath. At a particular time periods samples were taken and replaced with equivalent volume of fresh PBS-T. The steady state flux was determined by using the equation described in the literature [39].

### 2.7. Solubility

The solubility of pioglitazone in Duro-Tak 87-2516 patch with propylene glycol was determined by slide crystallization method. Briefly, required amount of drug (2–10% *w*/*w* of adhesive weight) was added to the adhesive with propylene glycol (5% *w*/*w*) in Eppendorf tube. The mixture was shaken for 48 h in a rotating stirrer (Remi, Mumbai, India) at room temperature. Few drops of mixture were placed on microscopic slides and dried at room temperature. The slides were then observed under an optical light microscope. 

### 2.8. Scanning Electron Microscopy (SEM)

Surface characteristics of DIA pioglitazone (6% *w*/*w*) patch with propylene glycol was recorded using SEM (457 V; Japan Electron Optics Laboratory, Tokyo, Japan). Photographs were captured by mounting the patch utilizing silver electrical tape and sputter coated (SCD005 Baltek Sputter Coater, Baltec AG, Balzers, Liechtenstein, Germany) with gold in presence of argon gas under reduced pressure.

### 2.9. In Vivo Evaluation

The Wistar male rats aged 6–8 weeks (weighing 200–250 g) housed in animal facility and fed on a standard pellet diet and water ad libitum was used. The guidelines of Institutional Animal Ethical Committee (MMCP/IEC/10/01) were strictly followed. Animals were indiscriminately distributed into two groups with six rats in individual group. Doral hair of the first group of rats were trimmed carefully using electric clipper and anesthetized by an intraperitoneal injection of phenobarbitone (30 mg/kg). The selected skin application area was washed (using saline) and cleaned with cotton swab prior to fixing the patch. The DIA patch of area 1.5 cm^2^ (equivalent to 22.5 mg of pioglitazone/rat) was applied on the dorsal area and lightly pressured by fingers to produce sufficient adhesion force so as to affix the patch to the skin membrane. The second group received pioglitazone suspension (4 mg/kg) and was administered via intra-gastric gavage. The oral dose of pioglitazone was calculated according to the standard human dose (45 mg) using the equation elaborated in the literature [40]. Blood samples, measuring ~200 µl, were collected at specified time intervals from the lateral tail vein in a dried heparinized tube. To minimize the drastic changes of central compartmental volume, an intraperitoneal injection of dextrose (250 μL) were administered to rats after every blood sampling. Protein precipitation of individual plasma samples (200 µL) was carried out with an equivalent ratio (1:1) of acetonitrile and 2-propanol by thorough mixing and vortexing for a period of 2 min. The supernatant fraction was injected (50 µl) to the HPLC column to analyze the amount of pioglitazone present in plasma. The sample collected at zero time was considered as the baseline value. A non-compartmental approach was implemented for estimating pharmacokinetic parameters like the area under the concentration-time curve from time 0 to ∞ (AUC_0–α_), maximum concentration achieved (*C*_max_), and the time to reach peak concentration (*T*_max_).

### 2.10. Skin Irritation Study

Local skin irritation of selected DIA patch was carried out in Wistar male rats with minor modification to the method reported [41]. Animals were divided into three groups with six animals in each. The hairs in the dorsal side were removed by trimming with an electric clipper and the treatment was done on 1.5 cm^2^ area, daily for three successive days. Group one was applied with standard skin irritant (2.5% sodium dodecyl sulphate), group 2 was applied with placebo transdermal patch and group 3 was applied with DIA patch. The area where the product applied was examined and graded for erythema/edema and scored based on Draize dermal scoring standards [42].

### 2.11. Data Analysis

Statistical analysis of data was carried out with independent t-test or ANOVA employing GraphPad Prism (version 5, Graphpad software, San Diego, CA, USA). *p* < 0.05 was treated as the level of significance.

## 3. Results and Discussion

### 3.1. Formulation of DIA Patches

DIA systems are widely used in transdermal therapy of drugs. It is a well-established fact that the adhesive layer of transdermal therapeutic system demonstrates a significant role in releasing besides controlling the delivery of therapeutic actives. Furthermore, the selection of acrylic pressure sensitive adhesive is crucial as it affects the drug permeation, stability and quality of the finished product. Similarly, drug concentration and skin permeation enhancers influence the delivery of drugs into and across the skin. Hence, the current study evaluated the effect of all these contributing factors while optimizing DIA pioglitazone patch. Acrylic adhesives were preferred over silicone and polyurethane pressure-sensitive adhesives, since they are more economical [43]. Hence, five acrylic pressure sensitive adhesives (Duro-Tak 87-2516, Duro-Tak 87-4287, Duro-Tak 87-900A, Duro-Tak 87-9301, Duro-Tak 87-4098) were chosen based on their physical properties and performance reported in the literature [44,45,46]. Acrylic pressure-sensitive adhesives, comprising carboxylic acid groups, were not checked since there is a possibility of reaction between amide or tertiary amine in pioglitazone and adhesive. The physical characteristics of adhesives used in the patches generally varies from each other with respect to the functional groups, existence or non-existence of vinyl acetate, crosslinkers, solid content and viscosity. The composition of prepared patches are outlined in Table 1. The prepared patches shown ideal organoleptic and physicochemical characteristics such as homogenous, soft and flexible. Moreover, the thickness of prepared DIA patches was in the range of ~1200–1500 µm.

### 3.2. Assay

The determination of drug content is an indispensable process quality control tool utilized in all dosage forms including transdermal system. Therefore, the drug content in prepared pioglitazone DIA patches were determined and summarized in Table 1. The results showed greater pioglitazone content (>95%) and were comparable in all the DIA patches studied (Table 1). The data here signifies the uniformity of drug content in all the developed patches and imply that the amount of drug is not affected by adhesive, chemical enhancers or pioglitazone concentrations, in the current experimental conditions.

### 3.3. In Vitro Release

**Figure 1:**
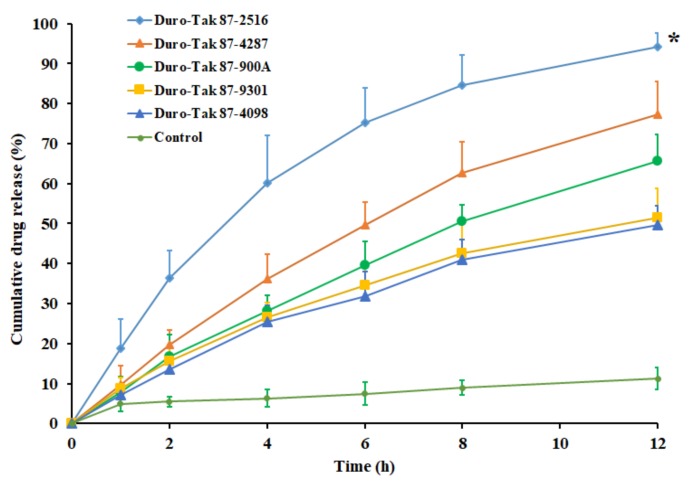
Comparison of the percentage of pioglitazone released at various time intervals from DIA pioglitazone (2% *w*/*w*) patch prepared using various adhesives. All values are mean ± SD (*n* = 6). The profile of DIA patch prepared using Duro-Tak 87-2516 adhesive is statistically (*) different (*p* < 0.001) when compared to other adhesives tested.

The release of pioglitazone from the DIA matrix is an essential step for its transport across the skin barriers to elicit therapeutic effect. The drug release rate from an adhesive matrix is mainly controlled by drug solubility along with diffusion coefficient in the adhesive. Accordingly, the amount of pioglitazone release from the transdermal matrix will influence the drug permeation across the skin membrane. The release of pioglitazone from the DIA patches, prepared with selected acrylic pressure sensitive adhesives, containing pioglitazone (2% *w*/*w*), was compared with a control (pure drug) in Figure 1. It is evident from the Figure 1 that the drug release profile of the DIA patch, prepared using Duro-Tak 87-2516 adhesive, was distinct and significantly higher than the other adhesives tested (*p* < 0.001). In addition, it can also be seen from the profile of Duro-Tak 87-2516 adhesive patch that more than 60% of drug release has happened in 4 h. Such release pattern could be advantageous as a higher amount of pioglitazone will be available on the skin surface for the percutaneous absorption during the initial period. Followed by the rapid release phase, there was steady release with approximately 75%, 85%, and 94% of drug released in 6 h, 8 h, and 12 h, respectively. The drug release profile observed here indicates the potential of the DIA patch, prepared using Duro-Tak 87-2516, in order to provide the extended release of pioglitazone on the skin surface, assuming that DIA patch will be in close contact with the skin for such long duration. Overall, the rapid as well as extended release demonstrated by the Duro-Tak 87-2516 adhesive patch may provide effective drug release on the skin surface. In contrast, the drug release from all other adhesives tested was comparable and relatively low, though increased over time (Figure 1). One can easily presume that the observed discrepancy in the drug release could be due to the difference in the physical characteristics of adhesives studied which includes functional group, crosslinkers, viscosity, and solid content. However, the release of pure drug was low and slow with approximately 11% dissolved in 12 h. The results of release kinetics of pioglitazone from prepared DIA patches are summarized in Table 2. The higher *r*^2^, minimum SSR and low F values observed in the Table 2 suggests that the release of pioglitazone from the all the adhesives tested followed Weibull model diffusion controlled mechanism. In addition, the n value observed (<0.5) with the adhesive signifies that the release of pioglitazone from prepared DIA patches is by Fickian diffusion.

### 3.4. Effect of Adhesives

**Figure 2:**
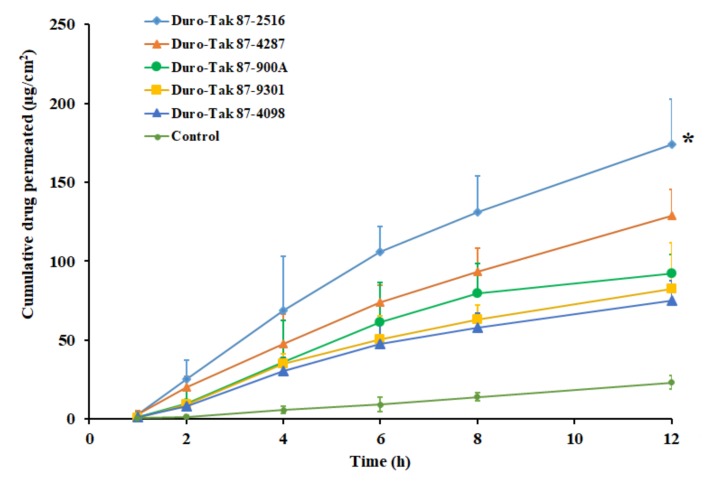
Amount of drug permeated across the rat skin membrane at different time intervals from various acrylic pressure sensitive adhesive pioglitazone (2% *w*/*w*) patches. All values are mean ± SD (*n* = 6). The profile of DIA patch prepared using Duro-Tak 87-2516 adhesive is statistically (*) different (*p* < 0.05) when compared to other adhesives tested.

The next stage of investigation assessed the effect of selected acrylic pressure-sensitive adhesives on the pioglitazone permeation. The concentration in the prepared DIA patches were fixed (2% *w*/*w*) and the study was carried out using the rat skin membrane as barrier. A control experiment was carried out with same concentration (3 mg/mL) of drug suspension in water. The amount of drugs, transported from various DIA patches, are illustrated in the Figure 2. The permeation parameters, observed with different adhesives, are outlined in Table 3. It is obvious from the Figure 2 that drug permeation increased with duration in all the adhesives tested. Greater drug permeation was noticed with DIA patch prepared using Duro-Tak 87-2516. The higher permeation of pioglitazone from the DIA patch with Duro-Tak 87-2516 could be corroborated with the release data observed in Figure 1. Certain quantity of pioglitazone was measured in the receptor medium during the initial sampling time (1 h) suggests fast permeation with short lag time, in all the adhesives tested (Table 3). It is also evident from Table 3 that the Duro-Tak 87-2516 exhibited greater transdermal flux (15.67 ± 2.35 µg/cm^2^/h) as compared to other adhesives tested. The permeability coefficient values decrease in the order; Duro-Tak 87-2516 > Duro-Tak 87-4287 > Duro-Tak 87-900A > Duro-Tak 87-9301 > Duro-Tak 87-4098 (Table 3). The variation in the permeability coefficient values, observed with DIA patches, can be directly correlated to the drug release profiles observed in Figure 1. It is also evident from Figure 2 that the drug permeation rate was moderately low after 6 h in Duro-Tak 87-900A, Duro-Tak 87-9301 and Duro-Tak 87-4098 adhesives. These low rates of drug permeation could also be correlated with the difference in the rate of pioglitazone release from the prepared DIA patches (Figure 1). On the other hand, the drug permeation from the control was slow and low (1.93 ± 0.87 µg/cm^2^/h) also could be due to low dissolution observed in Figure 1. Overall, the data revealed that the transdermal delivery of pioglitazone was influenced by the adhesives studied, under same conditions. The variation in composition and properties of acrylic pressure sensitive adhesives have resulted in difference in release rate which, in turn, influenced the skin permeation of pioglitazone. Duro-Tak 87-2516 is an acrylate copolymer constituting vinyl acetate is considered to be comparatively more hydrophilic in comparison to other adhesives. Therefore, it is assumed that the greater release of pioglitazone from patch might be probably because of the hydrophilic characteristics of the adhesive, albeit the exact delivery mechanism should be further examined. Due to the higher permeation profile, demonstrated by Duro-Tak 87-2516, it was chosen for further for ex vivo and in vivo investigations. 

### 3.5. Effect of Permeation Enhancers

**Figure 3:**
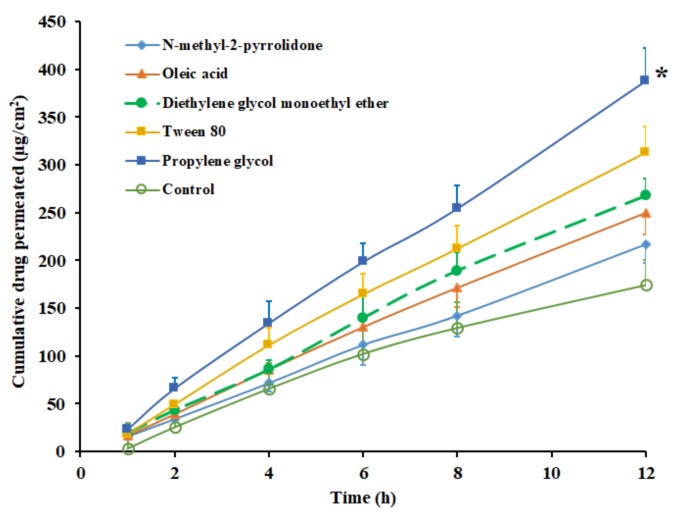
Amount of drug permeated across the rat skin membrane at different time intervals from Duro-Tak 87-2516 pioglitazone (2% *w/w*) patch with various skin permeation enhancers. All values are mean ± SD (*n* = 6). The profile of DIA patch with propylene glycol is statistically (*) different (*p* < 0.05) when compared to other enhancers tested.

The role played by permeation-enhancing agents in the transdermal delivery of drugs has been extensively investigated in the last few decades. In this context, the incorporation of such chemicals in DIA is demonstrated as a promising approach, which disrupts the skin membrane, overcomes the drug transport barriers and thereby improves the transdermal flux [47]. Consequently, to enhance the solubility, drug loading and the transdermal flux of pioglitazone, potent skin permeation enhancers were selected. Permeation enhancers, which are widely used and considered safe, such as NMP, diethylene glycol monoethyl ether, propylene glycol, Tween 80 and oleic acid were incorporated in the selected transdermal pioglitazone (2% *w*/*w*) patch, fabricated using Duro-Tak 87-2516. The permeation enhancer’s concentration was set at 5% *w*/*w* as disclosed in various studies [48]. The control experiment was carried out without an enhancer under the same experimental conditions. The permeation profiles of pioglitazone from DIA patches, with various chemical enhancers, are displayed in Figure 3, and the permeation parameters observed are detailed in Table 3. It can be understood from the Figure 3 that the incorporation of permeation enhancers increased the skin permeation of pioglitazone, but not to the same extent. The permeability coefficient decreased in the order; propylene glycol > Tween 80 > diethylene glycol monoethyl ether> oleic acid>NMP, among the enhancers tested (Table 3). The flux value observed from DIA patch with propylene glycol (32.59 ± 4.37 µg/cm^2^/h) accounts to ~2 folds (*p* < 0.0001) higher than the flux recorded with control (15.67 ± 2.35 µg/cm^2^/h). The enhancement in transdermal flux of pioglitazone with other permeation enhancers were ~1.7 folds (Tween 80), ~1.5 folds (diethylene glycol monoethyl ether), ~1.4 folds (oleic acid), and ~1.2 folds (NMP), when compared with control. The flux value improvement by these permeation enhancers could be related to their diverse transport mechanisms reported elsewhere [48,49]. The transdermal patch, comprising propylene glycol as an enhancer, was selected for further investigations, since it demonstrated a higher flux value over other tested enhancers.

### 3.6. Solubility

The solubility of pioglitazone in the selected DIA patch with propylene glycol was determined to incorporate the highest possible amount. Different concentrations of pioglitazone (2–10% *w*/*w* of adhesive weight) were added to the adhesive and the patches were prepared by casting it over glass slide. The prepared patches were observed frequently using optical microscopy to check the crystal formation on drying during storage at 25 °C. The saturation solubility of pioglitazone in Duro-Tak 87-2516 patch, with propylene glycol, is determined as the drug level which do not yield crystallization in patches during storage. The highest solubility of pioglitazone in Duro-Tak 87-2516 was found to be 6% *w*/*w* and the formulated patches did not exhibit any visible crystal development even after three months storage period at 25 °C. Nevertheless, when the drug concentration crossed the maximum limit (7% *w*/*w*), drug crystallization was observed after two months. Consequently, Duro-Tak 87-2516 pioglitazone patch with 2–6% *w*/*w* of drug content was further developed and the effect of drug content on permeation was evaluated. 

### 3.7. SEM

**Figure 4:**
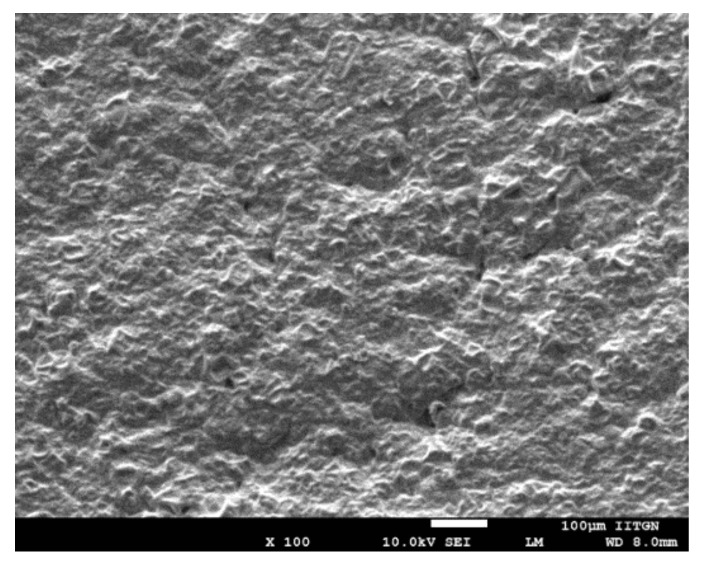
Representative scanning electron microscopy image of drug in adhesive pioglitazone (6% *w*/*w*) patch with propylene glycol.

High-resolution images, generated by SEM, can provide in-depth details about the shape and surface characteristics of the prepared DIA patch. It is noticeable from the image in Figure 4 that the transdermal patch is tortuous, porous, and free from any visible cracks. The irregular surface and pores observed in the transdermal patch are beneficial, as these would improve the skin breathability and water penetration in the prepared DIA patches, which in turn can lead to better patient compliance [50]. It is also obvious from the micrograph that the patch has good integrity. No evidence of drug crystals in the patches indicate the presence of drugs in the molecular form and are homogenously dispersed. Thus, the prepared DIA system possess necessary morphological features for transdermal application.

### 3.8. Effect of Drug Content

**Figure 5:**
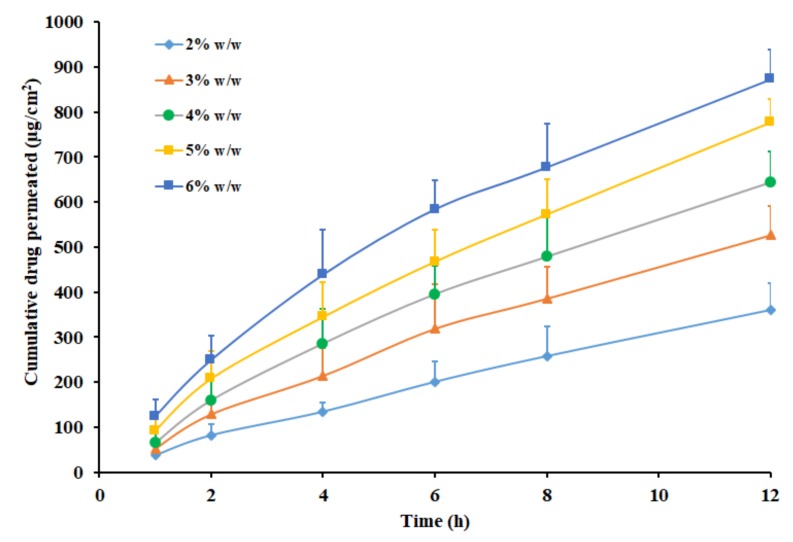
Amount of drug permeated across the rat skin membrane from Duro-Tak 87-2516 pioglitazone patch with propylene glycol at different drug level (2–6% *w*/*w*). All values are mean ± SD (*n* = 6).

The effect of pioglitazone content (2–6% *w*/*w*) on the transdermal flux was evaluated in the next phase, utilizing the patch prepared with Duro-Tak 87-2516 adhesive. Figure 5 exhibits that pioglitazone flux increased proportionately as the concentration increased during the entire study period (12 h). A direct relation between the pioglitazone content and the transdermal permeation was observed, in the current experimental condition (Figure 5). The permeation parameters observed with different drug concentrations are outlined in Table 3. It is apparent from the table values that the flux was greater when the drug concentration was high (6% *w*/*w*) and reduced with a decrease in drug concentrations levels. The observed enhancement in flux was ~2.2 folds when the pioglitazone level was elevated from 2% *w*/*w* to 6% *w*/*w*. One can easily correlate this observation with the proven fact that the thermodynamic activity reaches its peak, when the drug content level is at the saturation level in the matrix. Moreover, this interpretation is in full agreement with various studies reported earlier where the more drug content in the transdermal patch resulted in enhanced drug delivery [51]. The cumulative amount of pioglitazone delivered after 12 h was summarized in Table 3. The data indicate that the drug permeation was maximum when the pioglitazone in the patch is at 6% *w*/*w*, hence selected for in vivo studies.

### 3.9. In Vivo

**Figure 6:**
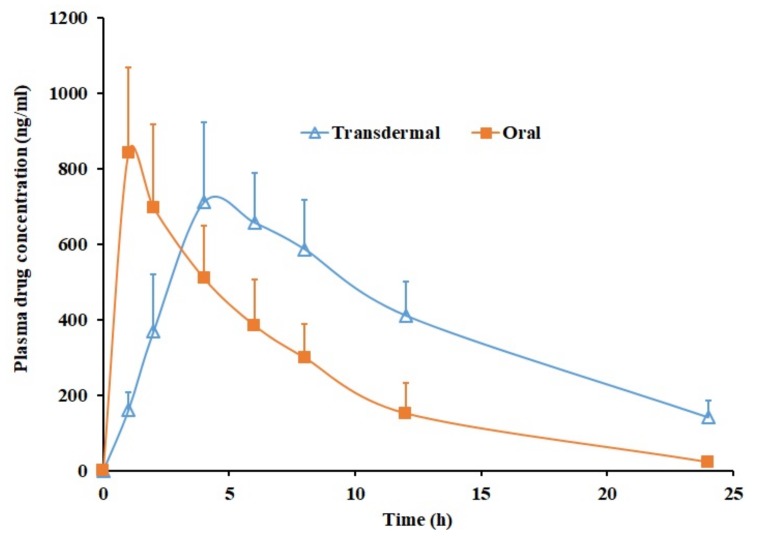
Plasma profiles of pioglitazone from transdermal Duro-Tak 87-2516 pioglitazone (6% *w*/*w*) patch with propylene glycol and oral administration (4 mg/kg). All values are mean ± SD (*n* = 6).

To hold the promise for clinical application, in vivo evaluations were performed in rats using Duro-Tak 87-2516 pioglitazone (6% *w*/*w*) patch with propylene glycol. The pharmacokinetics of transdermal therapy were compared with oral therapy as pioglitazone is an orally active antidiabetic used in chronic therapy. The oral dose of pioglitazone administered was 4 mg/kg, while DIA patch applied was approximately 22.5 mg/rat. Figure 6 correlates the average plasma concentration time profiles of pioglitazone obtained after transdermal and oral administration. The calculated pharmacokinetic parameters (C_max_, T_max_ and AUC_0–α_) are outlined in Table 4. It is clear from Figure 6 that the pharmacokinetic parameters computed for pioglitazone were distinct in case of transdermal and oral suspension. The pioglitazone permeation in transdermal therapy was slow in the initial hour (160.63 ± 47.81 ng/mL) while in oral therapy the C_max_ (842.30 ± 226.01 ng/mL) was attained quickly within one hour. It seems that the pioglitazone permeation from DIA patch continued to increase with time as the drug concentration in the plasma raised until 4 h (T_max_) with a C_max_ value of 711.83 ± 210.06 ng/mL, which is comparable with oral therapy (*p* = 0.3247). Later, the plasma level of pioglitazone slowly decreased until 24 h. The average value of AUC_0-α_ in transdermal application was ~2 times higher (*p* < 0.0001), as related to oral suspension (Table 4) indicates the enhancement in the pioglitazone bioavailability. The pioglitazone level achieved in the transdermal therapy seems very near to the effective therapeutic concentration level (~1 µg/mL). However, it should be emphasized that the area of the DIA patch used in this study is relatively small (1.5 cm^2^) and can be further increased in clinical situation to achieve greater drug plasma concentration. Overall, the data signifies the potential of developed DIA pioglitazone patch for the transdermal therapy in diabetic population. 

### 3.10. Skin Irritation

It is essential for the developed DIA transdermal systems to have good skin compatibility and no apparent skin irritation for its clinical use. The results of the skin irritancy test indicates a higher primary irritancy index (6.67 ± 0.82) with standard skin irritant, while relatively low value was observed in transdermal placebo patch (0.83 ± 0.75) and DIA patch (0.92 ± 0.66). In addition, no noticeable edema were visible on the rat’s skin of the placebo and DIA patches. These results signify that the placebo and DIA patches can be considered as non-irritants to the skin. 

## 4. Conclusions

A systematic investigation was carried out to develop DIA pioglitazone transdermal patch by assessing various parameters to provide effective delivery. The effect of formulation factors like pressure-sensitive adhesives, skin permeation enhancers, solubility were examined at various stages. The discrepancies in drug permeation with various patches can be correlated to variation in the pioglitazone release rate. Duro-Tak 87-2516 patch with propylene glycol showed higher transdermal flux, while increasing the drug level potentially enhanced the transdermal flux. The selected Duro-Tak 87-2516 pioglitazone (6% *w*/*w*) patch with propylene glycol was evaluated in rat model. Indeed, the conspicuous improvement in AUC by transdermal therapy implies better bioavailability of pioglitazone from the fabricated DIA patch. In conclusion, transdermal therapy of pioglitazone appears to be a promising substitute for its systemic delivery for the management of Type 2 diabetes, though to validate the in vivo efficacy a bioequivalence study need to be carried out in human.

## Figures and Tables

**Table 1:** Composition of prepared patches and the drug content.

Batch Code	Adhesive	Pioglitazone Incorporated (% *w*/*w*)	Enhancer (5% *w*/*w*)	Amount in 1 cm^2^ (mg)
F1	Duro-Tak 87-4098	2	-	4.7 ± 0.2
F2	Duro-Tak 87-9301	2	-	4.8 ± 0.1
F3	Duro-Tak 87-900A	2	-	4.7 ± 0.2
F4	Duro-Tak 87-4287	2	-	4.8 ± 0.1
F5	Duro-Tak 87-2516	2	-	4.7 ± 0.1
F6	Duro-Tak 87-2516	2	*N*-methyl-2-pyrrolidone	4.7 ± 0.1
F7	Duro-Tak 87-2516	2	Oleic acid	4.8 ± 0.2
F8	Duro-Tak 87-2516	2	Diethylene glycol monoethyl ether	4.7 ± 0.1
F9	Duro-Tak 87-2516	2	Tween 80	4.7 ± 0.2
F10	Duro-Tak 87-2516	2	Propylene glycol	4.8 ± 0.2
F11	Duro-Tak 87-2516	3	Propylene glycol	7.1 ± 0.3
F12	Duro-Tak 87-2516	4	Propylene glycol	9.6 ± 0.3
F13	Duro-Tak 87-2516	5	Propylene glycol	12.1 ± 0.4
F14	Duro-Tak 87-2516	6	Propylene glycol	14.5 ± 0.4

**Table 2:** Model fitting for drug in adhesive (DIA) pioglitazone patches with various adhesives.

Adhesive	Factors	Model Name					
		Zero Order	First Order	Higuchi	Korsmeyer-Peppas	Weibull Model	Hixson-Crowell
Duro-Tak 87-*4098*	*r* ^2^	0.9602	0.9877	0.9729	0.9905	0.9961	0.9804
	SSR	78.76	23.04	53.53	31.39	9.16	37.09
	FR	15.75	4.61	10.71	6.28	1.83	7.42
Duro-Tak 87-*9301*	*r* ^2^	0.9539	0.9866	0.9814	0.9935	0.9984	0.9777
	SSR	96.20	28.76	38.87	21.87	3.90	46.09
	FR	19.24	5.75	7.77	4.37	0.78	9.22
Duro-Tak 87-900A	*r* ^2^	0.9823	0.9990	0.9605	0.9932	0.9981	0.9986
	SSR	59.53	4.24	132.53	25.63	2.88	5.17
	FR	11.91	0.85	26.51	5.13	0.58	1.03
Duro-Tak 87-*4287*	*r* ^2^	0.9695	0.9983	0.9657	0.9919	0.9996	0.9978
	SSR	147.99	13.15	166.88	82.07	1.92	7.43
	FR	29.60	2.63	33.38	16.41	0.38	1.49
Duro-Tak 87-*2516*	*r* ^2^	0.8788	0.9989	0.9776	0.9648	0.9996	0.9834
	SSR	906.27	4.12	167.80	331.91	1.04	144.05
	FR	181.25	0.82	33.56	66.38	0.21	28.81

*r*^2^: Correlation coefficient; SSR: Sum of square of residuals; FR: Fischer ratio.

**Table 3:** Ex vivo permeation parameters observed in various treatments.

Batch Code	Lag Time (h)	Flux (µg/cm^2^/h)	Cumulative Amount Permeated (12 h) (µg/cm^2^)	Permeability Coefficient (cm/h × 10^−4^)
F1	0.18 ± 0.06	6.90 ± 1.55	74.83 ± 12.66	3.12 ± 0.94
F2	0.16 ± 0.05	7.54 ± 2.92	82.25 ± 29.20	3.43 ± 1.18
F3	0.17 ± 0.06	8.78 ± 3.14	92.12 ± 23.96	3.84 ± 1.05
F4	0.14 ± 0.04	11.42 ± 3.64	129.02 ± 20.42	5.38 ± 1.47
F5	0.09 ± 0.02	15.67 ± 2.35	174.24 ± 39.61	7.26 ± 1.24
F6	0.10 ± 0.04	18.27 ± 3.71	216.91 ± 38.94	9.04 ± 1.32
F7	0.09 ± 0.03	21.32 ± 3.86	249.86 ± 24.74	10.41 ± 1.81
F8	0.08 ± 0.03	22.95 ± 3.91	267.69 ± 32.45	11.12 ± 1.73
F9	0.07 ± 0.02	26.66 ± 4.18	313.01 ± 22.23	13.04 ± 1.90
F10	0.06 ± 0.01	32.59 ± 4.37	387.90 ± 33.08	15.16 ± 1.15
F11	0.06 ± 0.03	43.86 ± 4.92	526.28 ± 65.21	14.61 ± 1.95
F12	0.05 ± 0.02	53.66 ± 4.87	643.89 ± 68.17	14.97 ± 1.41
F13	0.05 ± 0.03	64.74 ± 5.83	776.86 ± 52.94	14.56 ± 2.95
F14	0.05 ± 0.02	72.68 ± 5.67	872.14 ± 67.36	14.91 ± 2.11

**Table 4:** Pharmacokinetic parameters of pioglitazone in plasma following transdermal and oral administration in rats. T_max_, time of maximum concentration; C_max_ indicates maximum concentration; AUC_0–α_, area under the plasma concentration-time curve.

Parameter	Transdermal	Oral Suspension
T_max_ (h)	4	1
C_max_ (ng/mL)	711.83 ± 210.06	842.30 ± 226.01
AUC_0–α_ (ng.h/mL)	13,506.51 ± 1649.92 *	6082.56 ± 1384.08

Data expressed as average ± SD (*n* = 6) and * *p* < 0.0001 were considered as significant.
